# Left-sided portal hypertension caused by a solid pseudopapillary neoplasm of pancreas tail: a pediatric case report

**DOI:** 10.1186/s40792-024-02034-1

**Published:** 2024-11-11

**Authors:** Toko Sihnkai, Kouji Masumoto, Yohei Sanmoto, Akio Kawami, Miki Ishikawa, Shunsuke Fujii, Tsukasa Saida, Toshitaka Ishiguro, Noriaki Sakamoto

**Affiliations:** 1 Department of Pediatric Surgery, Institute of Medicine, University of Tsukuba, 1-1-1 Tennodai, Tsukuba, Ibaraki 305-8575 Japan; 2 Department of Radiology, Institute of Medicine, University of Tsukuba, 1-1-1 Tennodai, Tsukuba, Ibaraki 305-8575 Japan; 3 Department of Diagnostic Pathology, Institute of Medicine, University of Tsukuba, 1-1-1 Tennodai, Tsukuba, Ibaraki 305-8575 Japan

**Keywords:** Solid pseudopapillary neoplasm, Pediatric, Left-sided portal hypertension, Splenic venous stenosis, Splenectomy

## Abstract

**Background:**

Solid pseudopapillary neoplasm (SPN) is a low-grade malignant tumor that occurs in 60% of all pediatric pancreas tumors. Radical tumor resection is essential; however, spleen preservation is also crucial to prevent overwhelming post-splenectomy infection. In contrast, spleen preservation is not always possible, because left-sided portal hypertension (LSPH) can cause splenic vein stenosis or occlusion induced by pancreatic tumor. We herein report on a pediatric patient of LSPH due to SPN in the pancreatic tail.

**Case presentation:**

A 12-year-old girl was admitted to our hospital with left upper quadrant abdominal pain. A solid mass was palpated in the left costal region. The patient showed slight anemia (Hb: 11.8 g/dL) and elevation of inflammatory reaction (CRP: 5.98 mg/dL) without positive tumor markers. A radiological examination revealed that a 9 cm-sized mass with hemorrhagic necrosis in the pancreatic tail. Splenic venous flow was not detected and collateral draining into the left gastric vein and left renal veins were developed with splenomegaly. LSPH was involved at the time of diagnosis. The tumor was diagnosed with SPN, hence tumor resection with spleen preservation was performed. Six months after surgery, the patient developed a left quadrant abdominal pain that worsened during exercise. There was no improvement of splenic venous flow and splenomegaly. LSPH remained with splenomegaly, which possibly triggered the patient’s abdominal pain. The patient underwent splenectomy 9 months after the tumor resection. After the splenectomy, the patient’s abdominal pain disappeared without any recurrence 8-year post-surgery.

**Conclusions:**

LSPH has not been a major focus in previous SPN pediatric patients, although most symptomatic LSPH patients required splenectomy. Careful post-operative observation for LSPH is important for pediatric SPN patients.

## Introduction

Solid pseudopapillary neoplasm (SPN) is a low-grade malignant tumor that occurs in 60% of all pediatric pancreas tumor patients [1]. Radical resection is essential to cure of SPN [1, 2], but spleen preservation is crucial to prevent a lifetime risk of overwhelming post-splenectomy infection (OPSI) [3]. However, spleen preservation may not be possible due to left-sided portal hypertension (LSPH) caused by splenic vein compression or occlusion induced by pancreatic tumor [4–6]. LSPH is defined as a rare entity of portal hypertension caused by splenic vein occlusion or stenosis with patent extra hepatic portal vein and normal liver function [7–10]. Blocked splenic venous flow drains via the short gastric, coronary, gastroepiploic veins [7–10]. The incident rates of LSPH are estimated at less than 5% of all cases involving portal hypertension. The main causes of LSPH are pancreatitis (17–60%) and tumors (9–35%) [8–10]. LSPH induces gastroesophageal varices and bleeding, abdominal pain, and splenomegaly [8–10]. Recently, LSPH is known as a long-term and life-threatening complication of post pancreaticoduodenectomy [11]. In most LSPH cases are considered to be asymptomatic. Therefore, LSPH has not been previously focused as complication of tumor resection in SPN [8–10].

To draw attention to the importance of LSPH as a potential but severe complications of pancreas tumor, we herein report on a pediatric patient who received a splenectomy due to LSPH after pancreas tail SPN resection.

## Case report

**Fig. 1:**
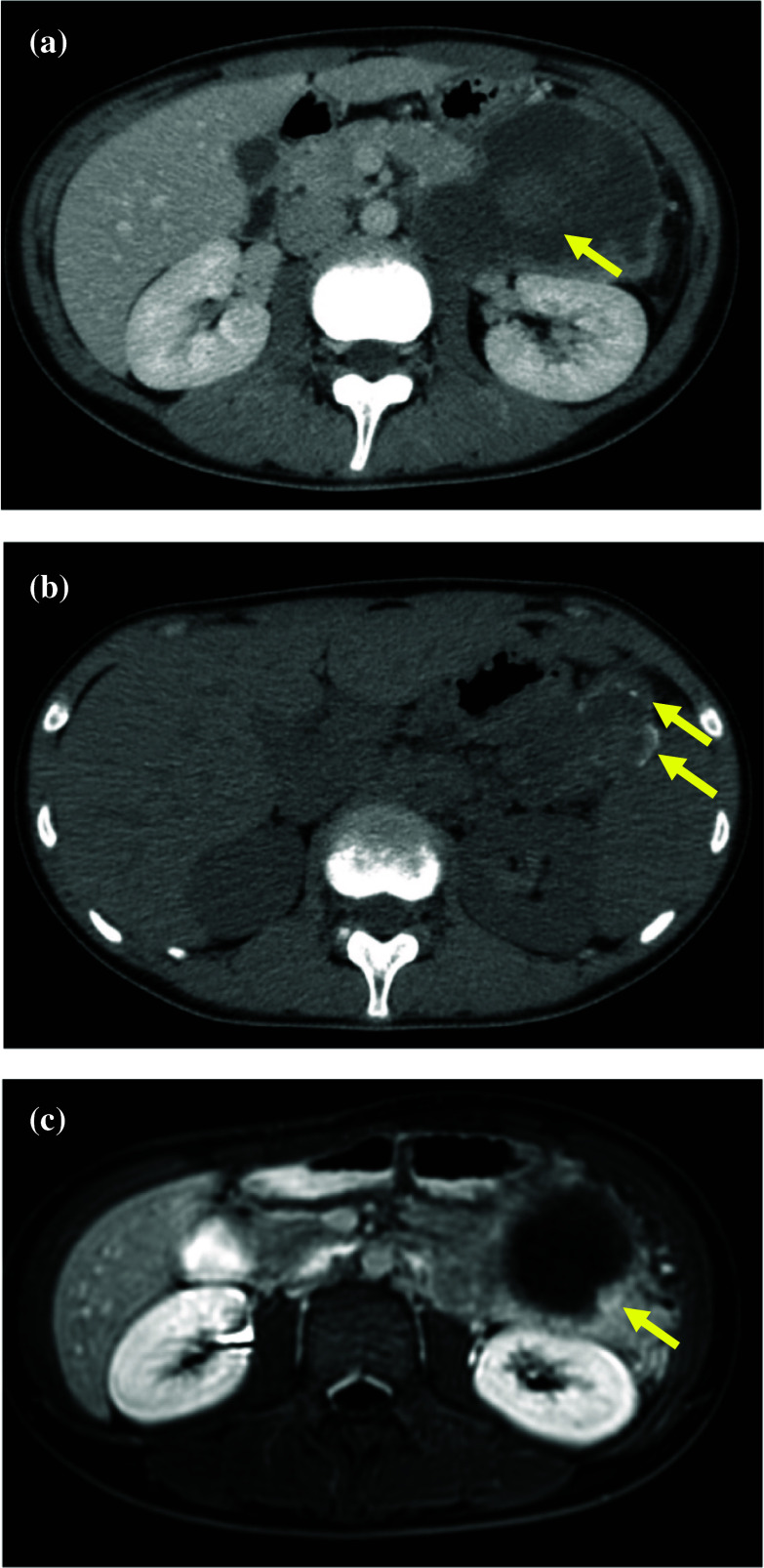
**a** Enhanced CT. An 8.3 × 5.3 × 7.1 cm cystic pancreatic tail tumor with hemorrhage (yellow arrow). **b** Plane CT. The pancreatic tail tumor partially contained calcifications (yellow arrow). **c** MRI T2-weighted image. A nodular enhancement inside the mass was suggested a high cellular density lesion (yellow arrow)

**Fig. 2:**
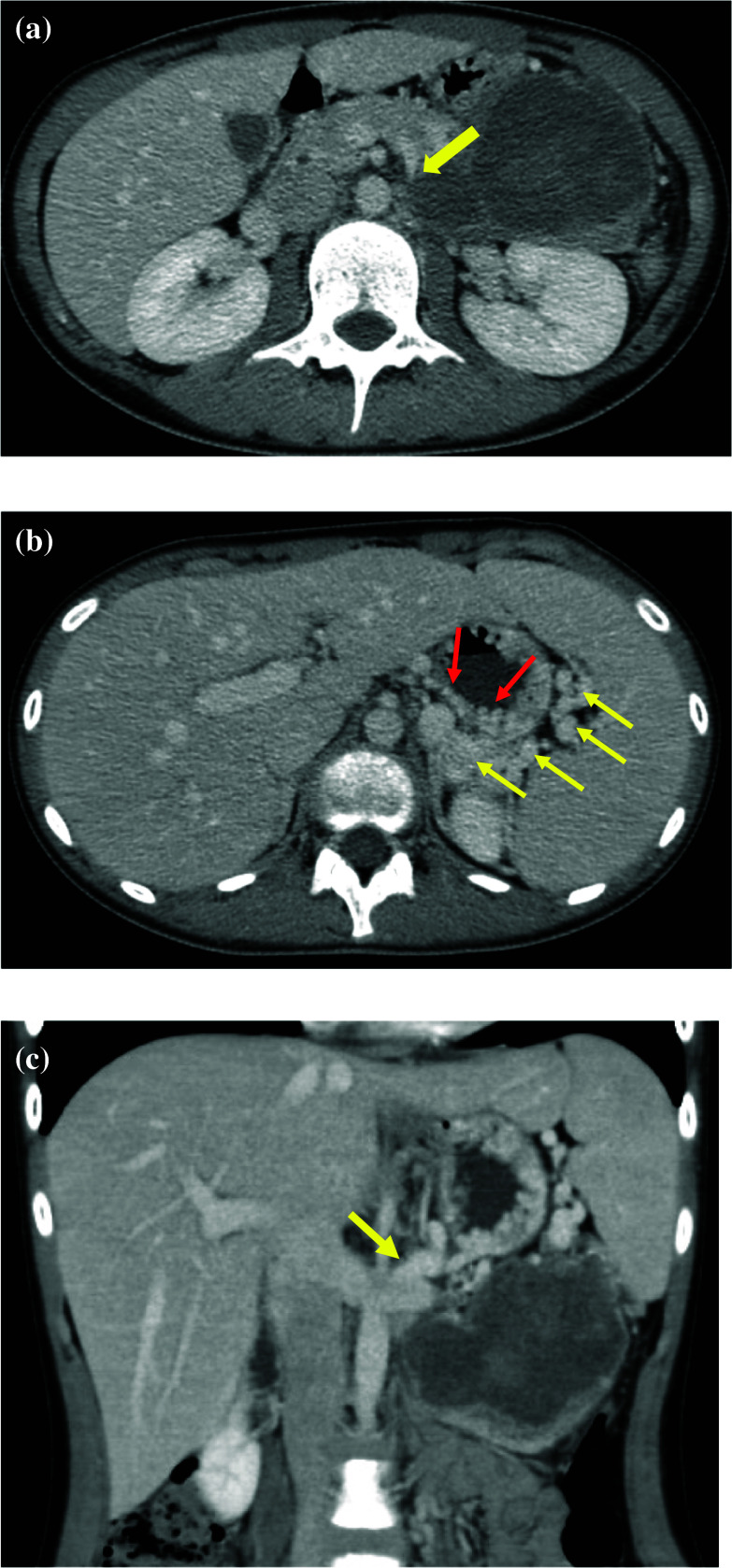
**a** Enhanced CT. **a** Shows that the splenic vein was narrowed like as beak due to compression and stretch by the tumor. **b** Enhanced CT. Collateral pathways developed through a short gastric vein and the left gastric vein (yellow arrow). Varices were developed in the gastric wall (red arrow). **c** Enhanced CT. A large spleno-renal shunt was developed (yellow arrow)

A 12-year-old girl was admitted to our hospital for left upper quadrant abdominal pain with an elevated fever. These symptoms continued for 7 days. The patient had no past medical histories except for a latex allergy. A physical examination revealed a fist-sized solid mass in the left costal region. The patient had spontaneous pain and tenderness on and around the mass. The patient’s vital signs were BT: 37.5 °C, BP: 92/62 mmHg, HR: 62/min, RR: 16/min. Except for slight anemia (Hb: 11.8 g/dL) and elevation of CRP level (5.98 mg/dL), her laboratory data was normal, and the levels of tumor makers were also within the normal range. Contrast-enhanced computed tomography (CT) revealed a well-circumscribed cystic and solid mass with hemorrhage measuring 8.3 × 5.3 × 7.1 cm in the pancreatic tail (Fig. 1a). The mass contained some calcifications in plain CT (Fig. 1b). T2-weighted magnetic resonance images (MRI) showed a nodular enhancement inside the mass suggesting a high cellular density lesion (Fig. 1c). According to the radiological findings, the mass was diagnosed as SPN of the pancreatic tail. It was difficult to trace the main splenic vein at the splenic hilum, but at the dorsal part of pancreas, the splenic vein was narrowed like as beak in enhanced CT images (Fig. 2a). This finding was considered that the splenic vein was compressed and stretched by the tumor. There was no splenic venous thrombosis or tumor invasion to the splenic vein.

Abdominal ultrasound sonography (US) revealed that the spleen was enlarged (12.3 × 5.2 cm). Abdominal US and contrast-enhanced CT showed that multiple collateral pathways of short gastric veins and left gastric veins (Fig. 2b) with a large spleno-renal shunt had developed (Fig. 2c). These findings indicated left-sided portal hypertension (LSPH) due to tumor compression and stretch of splenic vein. LSPH and splenomegaly had already been observed at the time of diagnosis before the tumor resection. We discussed the possibility of spleen preservation at the time of surgery to prevent OPSI and decline LSPH after tumor resection. We concluded that splenic venous flow was resumed by tumor resection improving splenic vein stenosis caused by the tumor compression and stretch. Therefore, we chose the tumor resection with spleen preservation to prevent OPSI. We performed tumor enucleation by open surgery, the pancreas tail was partially resected due to severe adhesion. The stump of resected pancreas was closed by running suture. During the procedure, many dilated collateral splenic veins were seen. Some of the dilated collateral splenic veins were resected to detach the tumor.

**Fig. 3:**
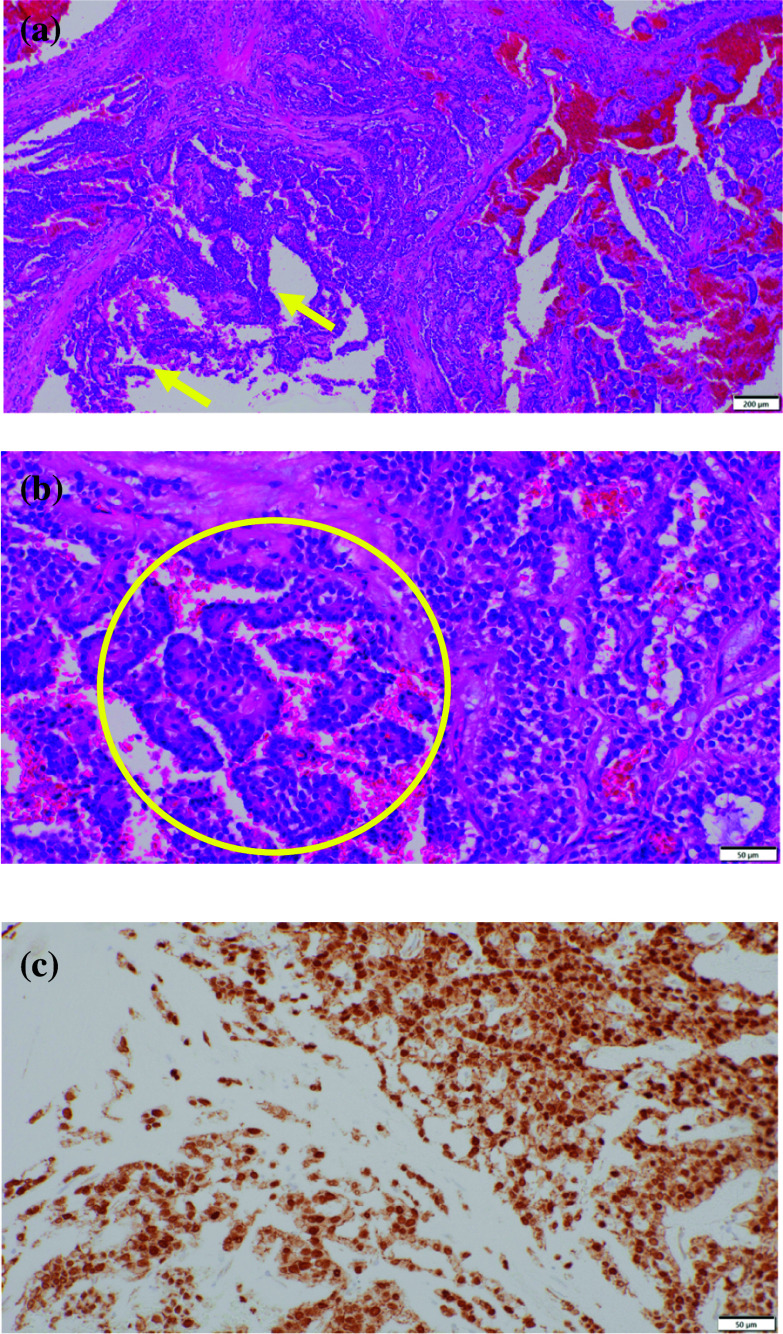
**a**, **b** H&E. The tumor consisted of solid lesions with pseudopapillary lesions (yellow arrow). Pseudo-rosette structures were seen (yellow circle). **c** Immuno-histochemical findings. The tumor nuclei were positive in β-catenin staining

Histological findings showed that the tumor consisted of solid lesions with pseudopapillary features and pseudo-rosette structures (Fig. 3a, b). Immuno-histochemical findings revealed that the tumor nuclei were positive in β-catenin (Fig. 3c). Based on those histological findings, the tumor was diagnosed SPN.

**Fig. 4:**
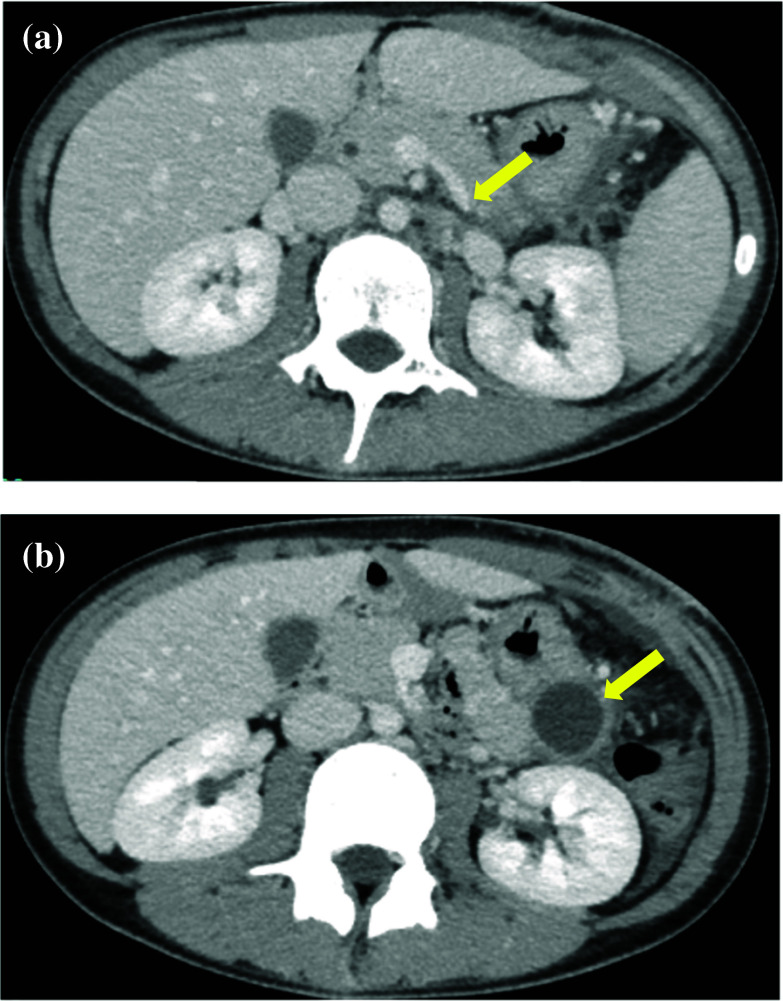
**a** Enhanced CT. Splenic vein stenosis remained at POD 13 (yellow arrow). **b** Enhanced CT. A cyst of POPF revealed at the stump of pancreas tail at POD 13 (yellow arrow)

**Fig. 5:**
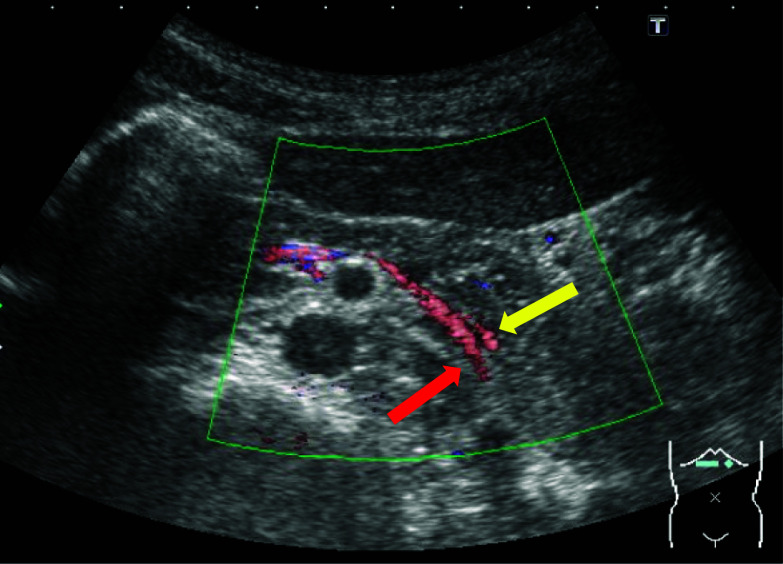
Abdominal US. A detailed anatomy of splenic vein and inferior mesenteric vein (red arrow) were revealed, the splenic venous flow (yellow arrow) at the dorsal part of pancreas was supplied through the pancreas parenchyma

The patient was started enteral nutrition postoperative day (POD) 4, the drain discharges were 10–25 mL. The drain was clumped at POD 5. US showed no fluid retention around the pancreas tail, the drain was removed at POD 6. The patient had left lateral abdominal pain at POD 8 and the fluid retention around the pancreas tail was detected by US at POD 10. The patient did not show fever elevation. The laboratory data showed WBC: 3200, Amylase: 245 IU/L, Lipase: 203 IU/L, CRP: 3.07. Postoperative pancreatic fistula (POPF) was occurred. We started conservative treatment such as total parental nutrition and ulinastatin. Enhanced CT at POD 13 showed the splenic vein stenosis (Fig. 4a) and pancreatic tail cyst (Fig. 4b). The patient’s abdominal pain gradually improved and disappeared in 10 days without any infectious signs. The pancreas tail cyst was disappeared POD 29 by US without drainage. Moreover, a detailed anatomy of splenic vein and inferior mesenteric vein were revealed, the main tract of splenic vein did not detect but, the splenic venous flow at the dorsal part of pancreas was supplied through the pancreas parenchyma (Fig. 5). It suggested that the main tract of splenic vein was obstructed at the splenic hilum and the main tract of splenic flow did not improve after tumor resection. Although International Study Group of Pancreatic Surgery grade B POPF was occurred, the patient was discharged on POD 47.

The patient had no symptom, LSPH was remained after discharge by regular check-up. Six months after surgery, the patient developed a left quadrant abdominal pain that worsened during exercise. As a result, the patient could not attend school due to severe abdominal pain. Abdominal US showed that the splenic vein stenosis did not improve after tumor resection and LSPH with splenomegaly (spleen was measured by 12.7 × 47 cm) remained. Although laboratory data: (WBC 4200/μL, RBC 4.29 × 10^6^/μL, and Plt 18.9 × 10^4^/μL) showed that the hypersplenism was not seen in this patient, her abdominal pain was supposed to cause by splenomegaly induced by LSPH. Splenectomy was performed 9 months after the tumor resection. After the splenectomy, her abdominal pain disappeared and there has been no recurrence over the past 8 years. The patient received preoperative pneumococcal vaccination and postoperative prophylactic antibiotics for 2 years. In addition, the patient has continued to receive pneumococcal and meningococcal vaccination every 5-year post-splenectomy.

## Discussion

LSPH is rare, but a possible pre- or post-operative complication of pancreas tumor occurs even in pediatric cases. However, there are no suggestion to LSPH as a complication of pediatric SPN in review reports [1, 2], therefore the accurate incidence rate of LSPH in pediatric SPN is not clear. There are three case reports of young–adult SPN aged 17-, 28-, and 19-year-old cases with LSPH at the time of diagnosis, who had received splenectomy during tumor resection [4–6]. It should concern the way of spleen preservation during the tumor resection who demonstrated LSPH pre-operatively, and laparoscopic pancreatectomy incorporating with the Warshaw technique [12] may be applicable. Although upper gastrointestinal varices are the main manifestation of LSPH in adult patients, the incidence of bleeding in gastrointestinal varices is considered rare due to adequate low-pressure collateral flow development [8–10]. But upper gastrointestinal varices bleeding can be a critical complication [11], close observation of LSPH is important as a post-operative complication. If symptoms of LSPH become apparent, splenectomy or selective embolization of the splenic arteries is the main treatment for adult LSPH [7–11]. Aggressive and routine vaccination post splenectomy [3] is necessary for patients of all ages.

## Conclusions

Previously, LSPH has not been a major focus in pediatric patients, because LSPH is an unrecognized complication of pancreas tumor. In our patient, abdominal pain due to LSPH was serious and might interfere with a patient’s daily life. Therefore, we should aware of LSPH and consider the treatment strategy of LSPH. If the symptom is serious as shown in our patient, the aggressive therapy for LSPH should be considered even after tumor resection.

## Data Availability

The data that support the findings of this report are available from the corresponding author upon reasonable request.

## Abbreviations

BT: Body temperature
BP: Blood pressure
HR: Heart rate
RR: Respiratory rate
SPN: Solid pseudopapillary neoplasm
OPSI: Overwhelming post-splenectomy infection
LSPH: Left-sided portal hypertension
CT: Computed tomography
MRI: Magnetic resonance images
US: Ultrasound sonography
POD: Post-operative day
POPF: Postoperative pancreatic fistula
